# Chronic neuromuscular electrical stimulation improves muscle mass and insulin sensitivity in a mouse model

**DOI:** 10.1038/s41598-019-43696-4

**Published:** 2019-05-10

**Authors:** Adiel Lotri-Koffi, Marion Pauly, Emeline Lemarié, Diane Godin-Ribuot, Renaud Tamisier, Jean-Louis Pépin, Isabelle Vivodtzev

**Affiliations:** 1 Univ. Grenoble Alpes, Inserm 1042, CHU Grenoble Alpes, HP2 Laboratory, Grenoble, France; 2 Cardiovascular Research Laboratory, Spaulding Rehabilitation Hospital, Cambridge, Massachusetts USA; 3 Department of Physical Medicine and Rehabilitation, Harvard Medical School, Boston, Massachusetts USA

**Keywords:** Endocrinology, Rehabilitation

## Abstract

Muscle wasting reduces functional capacity and increases cardiometabolic risk in chronic disease. Neuromuscular electrical stimulation (NMES) of the lower limb has been shown to reverse muscle wasting in these patients but its effect on cardiometabolic health is unclear. We investigated a mouse model of *in-vivo* non-invasive chronic NMES on muscle mass, insulin sensitivity and arterial blood pressure (BP). Twenty-three C57BL6 mice underwent unilateral NMES or sham training over 2.5 weeks while anesthetized by isoflurane. Lower limb muscle mass and the stimulated limb to non-stimulated limb muscle mass ratio were compared between groups (NMES vs. sham). Insulin sensitivity was assessed 48 h after training using an intraperitoneal insulin tolerance test (ITT) and BP was assessed before and after training using the tail-cuff technique. After training, muscle mass increased in NMES vs. sham (416 ± 6 vs. 397 ± 6 mg, p = 0.04) along with the ratio of muscle mass (+3 ± 1% vs. −1 ± 1% p = 0.04). Moreover, insulin sensitivity improved in NMES vs. sham (average blood glucose during ITT: 139.6 ± 8.5 vs. 161.9 ± 9.0 mg/dl blood, p = 0.01). BP was decreased in both groups, although it is likely that the effect of NMES on BP was dampened by repetitive anesthesia. The metabolic benefit of NMES training could be of great utility in patients with chronic disease. Moreover, the *clinical-like* mouse model of NMES is an effective tool to investigate the systemic effects of local muscle strengthening.

## Introduction

Muscle wasting is a common characteristic in chronic disease, due to systemic inflammation and whole-body protein turnover^1–3^. One consequence is a synergistic effect with ectopic fat accumulation augmenting cardiometabolic risk factors such as hypertension, dyslipidemia and arterial stiffness. This has well been described in chronic obstructive pulmonary disease (COPD)^4–6^, for example. In fact, there is a crosstalk between fat and muscular tissues. Mitochondrial defects in muscle of diabetic patients accelerate lipid deposition in non-adipose tissues and worsen insulin resistance^7^. Hence, muscle wasting not only reduces functional capacity of patients with advanced chronic disease, but also increases cardiometabolic morbidity and mortality. Therefore, maintaining skeletal muscle function has become an important therapeutic target to reduce cardiometabolic risk in chronic disease^7,8^.

Exercise training is currently the most available intervention to address muscle wasting^9,10^. However, patients with chronic disease may be too weak or too dyspneic to benefit from exercise training^11^ leading to a substantial proportion of patients (30–50%) who remain sedentary in the long term^12,13^. In addition, endurance training may insufficiently increase muscle mass^14^. To preserve muscle mass and function in patients with chronic disease and exercise limitations, neuromuscular electrical stimulation (NMES) has been previously used with success for more than a decade now^15^. This technique consists of intermittent electrical stimuli to generate muscle contractions via surface electrodes on the skin. More recently, studies have suggested that NMES could also be relevant for obesity and type II diabetes mellitus, provided it improves glucose metabolism^16–20^. For now, the most consistently reported adaptations after NMES training in patients with advanced chronic disease are increases in muscle strength (~30–50%) and in muscle mass or circumference (~6%) of the stimulated muscle^21,22^. However, these muscular adaptations might transfer into broader metabolic benefits. For example, patients with cystic fibrosis who performed NMES training before ergocycle training showed both larger quadriceps circumference and better endogenous secretion of insulin than those having performed ergocycle only^23^. In fact, a single session of NMES may enhance glucose uptake in patients with Type 2 Diabetes^16^. Of note, we have previously demonstrated that NMES training may improve muscle cross sectional area (CSA) by activating the insulin-like growth factor-1 (IGF-1)/AKT/mTOR signaling pathway in patients with COPD^24^. Since glucose translocation is known to be regulated by the IGF-1/Akt/AS160 signaling pathway^25^ and increased by resistance exercise via this specific pathway^26^, it seems reasonable to think that NMES might concurrently improve muscle mass and insulin sensitivity.

Hence, the impact of local muscle strengthening on metabolic outcomes (glucose tolerance, insulin sensitivity) remains incompletely elucidated and could be underestimated in humans. In fact, very few studies have investigated the systemic effects of chronic NMES training. Animal studies offer broad access to research investigations including blood, vessel, organs and nerves with easier tissue access. However, most animal studies in the current literature use NMES parameters that have no realistic application to clinical practice such as *in-situ* nerve stimulation^27,28^, *in-vitro* muscle stimulation^18,19^ or excessive duration of stimulation (several hours/day)^28,29^. To our knowledge, only the study of Ambrosio *et al*. employed an interesting *clinical-like* model of *in-vivo* chronic stimulation in mice, but the results were only preliminary and not well controlled^30^.

We contend that an *in-vivo* and chronic model of NMES in rodents using *realistic* parameters of stimulation (neuromuscular and <1 h per day) is necessary to better understand the actual systemic effect of NMES therapy. In the present study, based on the model of Ambrosio *et al*.^30^, we investigated a chronic (2.5 weeks) and non-invasive *in-vivo* stimulation model of the neuromuscular junction in mice that mimics the clinical use of NMES therapy. Therefore, the present study reports controlled (vs. sham) pilot results aiming to constitute a methodological basis for future research on the effects of local muscle protein growth on cardiometabolic health. As indicators of NMES efficacy, we assessed the mass of 4 different lower limb muscles (in both stimulated vs. non-stimulated limb), insulin sensitivity as a marker of metabolic impact, and arterial blood pressure as a marker of cardiovascular impact.

## Results

### Tolerance to repetitive Isoflurane anesthesia

**Figure 1:**
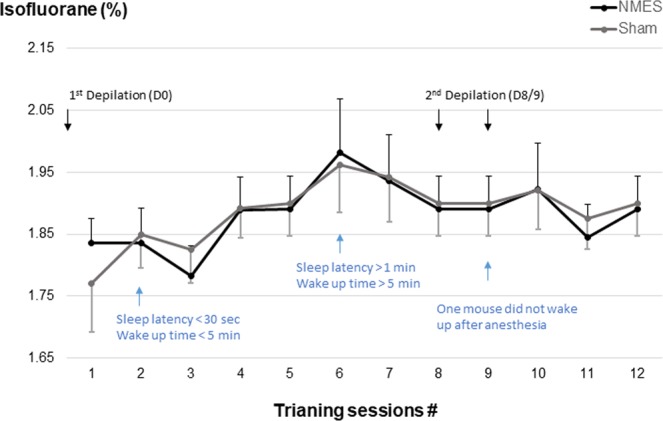
Concentration of isoflurane (%) used across the training sessions for both sham (grey dots and line) and NMES (black dots and line) groups.

Tolerance to isoflurane inhalation was good across the five first sessions with a rapid sleep latency (<30 sec) and a short wake up time (<5 min) (Fig. 1). After the 5^th^ session, higher levels of isoflurane were necessary to insure full anesthesia of the mice. In addition, sleep latency and wake-up time were both increased. Nevertheless, the mortality rate was very low over the whole period of the study. Only one mouse in the NMES group did not wake up after the 9^th^ training session under anesthesia. The average level of isoflurane needed to obtain a short but complete anesthesia during the whole period of training was similar between group (NMES: 1.88 ± 0.01 vs. sham: 1.89 ± 0.01%, p = 0.86).

### Body weight

**Figure 2:**
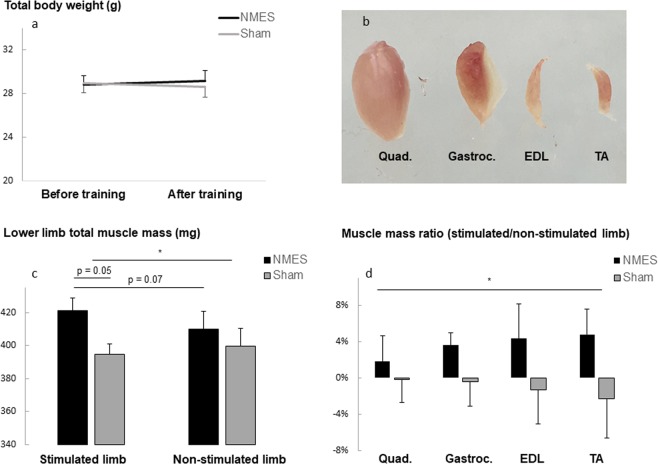
Effect of NMES training on total body weight (**a**), lower limb muscle mass (**c**) and on the stimulated limb to non-stimulated limb muscle mass ratio, depending on the different muscle groups of the lower limb (**b**,**d**). Values are means +/− SE (n = 23). *Two ways analyze of variance (Anova), p < 0.05.

Total body weight was not significantly different in the two groups of mice (NMES vs. sham) at baseline and at the end of the training period (p > 0.60). Changes in body weight over time (before vs. after training) tended to be different between groups however (NMES: +1.2 ± 0.9% sham: −1.1 ± 1.1%, p = 0.11) (Fig. 2a).

### Muscle mass

The total muscle mass of the lower limb was defined as the sum of 4 muscle masses of the lower limb, including the tibialis anterior (TA), the extensor digitalis lateralis (EDL), the gastrocnemius (Gastroc.) and the quadriceps (Quad.) (Fig. 2b). The total muscle mass was compared between groups (NMES vs. sham) and conditions (stimulated vs. non -stimulated limb). NMES training led to a significant increase in the total lower limb muscle mass compared to sham (group effect, 416 ± 6 vs. 397 ± 6 mg, p = 0.04, Fig. 2c). In addition, the muscle mass of the stimulated limb was higher in the NMES group than in the sham group (p = 0.05) and tended to be higher than the non-stimulated limb (p = 0.07) (Fig. 2c). Furthermore, to account for the influence of body weight change independently of NMES, we also compared the stimulated limb to the non-stimulated limb muscle mass ratio in each group (Fig. 2d). The ratio was improved in the NMES group compared to sham (+3 ± 1 vs. −1 ± 1%, p = 0.04) (Fig. 2d), suggesting that changes in muscle mass were not due to a change in total body weight. Despite a propensity to be higher in the agonist stimulated-muscle (TA and EDL), the ratio of muscle mass (stimulated limb vs. non-stimulated limb) was not significantly different between the 4 types of muscle (TA: +4.8 ± 2.8%, EDL: +4.4 ± 3.8%; Gastroc.: 3.6 ± 1.4% vs. Quadriceps: 1.8 ± 2.8%, p = 0.86).

### Metabolic effect of NMES

**Figure 3:**
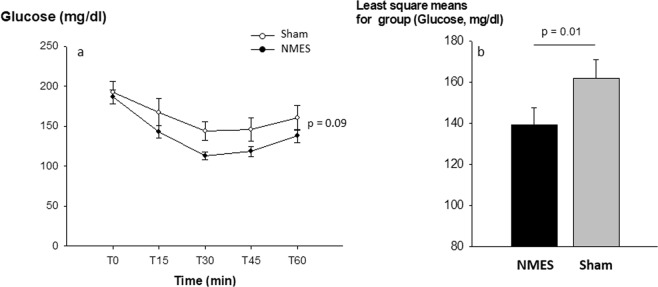
Effect of NMES training on insulin sensitivity as assessed by blood glucose concentration over time (**a**) and as an average (Least square means) (**b**) during the Intraperitoneal Insulin Tolerance Test. Values are means +/− SE (n = 16).

**Figure 4:**
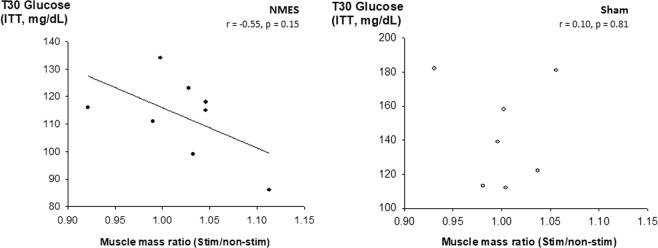
Relationship between muscle mass ratio (stimulated to non-stimulated leg ratio) and blood glucose concentration at T30 during the ITT in mice treated with NMES (**a**) or sham (**b**) training.

At baseline, after a 5 h fast, NMES and sham mice had similar levels of blood glucose. In contrast, dynamic tests exhibited a better response to insulin injection in the NMES group compared to sham. Indeed, there was a tendency of a lower level of blood glucose over time (p = 0.09, Fig. 3a) with a main effect at T30 and T45. In addition, the least square means were significantly lower in NMES vs. sham (139.6 ± 8.5 vs. 161.9 ± 9.0 mg/dl blood, p = 0.01, Fig. 3b) suggesting a higher level of insulin sensitivity in the NMES group. Moreover, we found a trend to significant relationship between glucose concentration at T30 and the stimulated to non-stimulated limb muscle mass ratio in mice treated with NMES (r = −0.55, p = 0.15). No relationship was found in the sham group (Fig. 4).

### Cardiovascular effect of NMES

**Figure 5:**
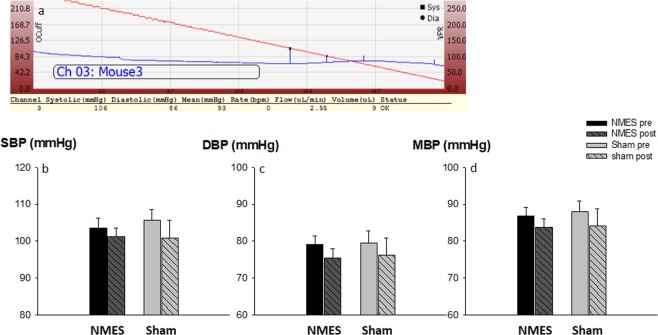
Effect of NMES training on arterial blood pressure. Individual example of a blood pressure measurement in mice. (**a**) Comparison of pre (plain) vs. post-training (hatched) values of systolic arterial pressure (SAP) (**b**), and diastolic arterial pressure (DAP) (**c**), in mice receiving NMES or sham training. Values are means +/− SE (n = 23).

**Figure 6:**
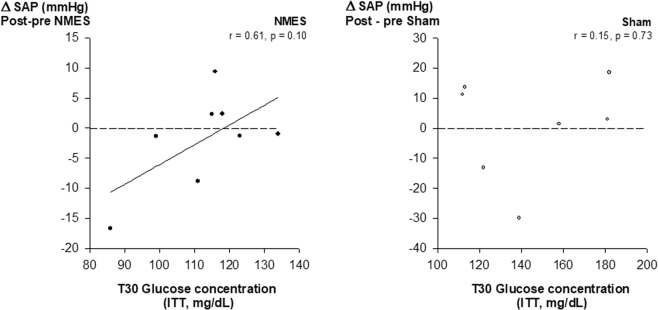
Relationship between blood glucose concentration at T30 during the ITT and changes in systolic blood pressure after training in mice treated with NMES (**a**) or sham (**b**) training.

Blood pressure was assessed noninvasively using the tail-cuff technique (Fig. 5). At baseline, systolic and diastolic blood pressures were similar between groups (NMES and sham). After the training period, systolic, diastolic and mean blood pressures were slightly lower in both groups with no significant difference between groups (p > 0.75, Fig. 5). However, we found a trend to significant relationship between glucose concentration at T30 (during ITT) and systolic blood pressure level in mice treated with NMES (SAP: r = 0.61, p = 0.10). No relationship was found in the sham group (p = 0.73) or in the whole group of mice (p > 0.40) (Fig. 6).

## Discussion

Our results suggest that a program of NMES training representing clinical practice (30 min per day, 5 days/wk., 2.5 weeks) is effective in improving both muscle mass and insulin sensitivity in mice, suggesting a general metabolic effect of local muscle strengthening. In addition, we found that metabolic improvements tended to transfer into reduction in systolic blood pressure, suggesting an overall cardiometabolic benefit of NMES. Lastly, the use of anesthesia during training sessions did not lead to excessive death in the present study and did not seem to limit NMES training. However, repetitive anesthesia may have reduced blood pressure over time in both groups, and potentially dampened the effect of NMES on blood pressure.

Compared to sham, NMES training induced an increase in muscle mass in mice by ~3% (Fig. 2). Although small, this increase in muscle mass occurred after only 12 sessions (2.5weeks) of training and in agreement with previous findings. For example, we previously reported an increase of ~6% in the cross sectional area after 6 weeks of NMES training in patients with COPD^24^ and Gondin *et al*. reported a similar level of  increase in muscle mass after 8 weeks of NMES training in healthy young subjects^31^. Hence, it is likely that longer exposure would lead to greater improvements, but nonetheless a 2.5-week NMES program is sufficient to induce muscle adaptations in mice. An intriguing result is the lack of significant difference in changes in muscle mass between the different type of muscle of the lower limb (Fig. 2d). Indeed, since dorsiflexion and full extension of the digits are known to be consequences of TA and EDL muscle contractions (i.e. stimulation) respectively, we mostly expected increases in muscle mass of those 2 muscle groups. We did not expect however that antagonist muscles (gastrocnemius) or remote muscle from the site of stimulation (quadriceps) would also show adaptations to NMES. To our knowledge, no studies have previously reported muscle mass or weight of non-stimulated muscles after NMES training. However, the small but noteworthy increase in quadriceps and gastrocnemius muscle mass suggests that adaptations also occurred in proximal muscles with training. Considering that changes in muscle mass were controlled by the non-stimulated limb and by a sham group, the probability that this result was obtained by chance is low. It is also unlikely that co-contractions occurred with stimulation since we visually controlled for leg movement throughout the training sessions. However, muscle growth was lesser further from the site of stimulation (+4–5% for TA and EDL vs. +3.5% for Gastrocnemius and +2% for Quadriceps, respectively, Fig. 2d), suggesting that this effect was possibly related to propagation of the electrical current to proximal muscles such as Gastroc. and Quad. In fact, improvement in muscle strength of both stimulated (plantar flexor) and non-stimulated (dorsiflexor) antagonist muscles have been previously reported in humans after NMES training^32^. Hence, it is possible that muscle plasticity occurred proximally to the site of stimulation. For example, the overall movement of the limb (due to stimulation) may have led to mechanical deformation converted into signaling events via *phosphoproteomic* molecules that drive increase in muscle mass^33^. More studies are needed to define this phenomenon in subgroups of muscles that are not directly stimulated but proximal to stimulation sites. It is possible that our model of stimulation could be used to investigate muscle plasticity secondary to neuromuscular stimulation.

Aside from muscular improvements, there is growing interest for potential metabolic effects of NMES training in chronic disease, particularly in metabolic disease^34^. NMES training was recently shown to increase glucose infusion rate during euglycemic hyperinsulinemic clamp tests, suggesting an increase in insulin sensitivity after only 1 week of training in type 2 diabetes mellitus^35^. In addition, using high intensity and high volume of training (1 h × 6 times weekly for 8 weeks), NMES may also reduce Hemoglobin A1c in patients with type 2 diabetes mellitus^17^. In fact, glucose uptake has been enhanced in a dose dependent way during a single NMES session in patients with type II diabetes^16^. Although not well-controlled, these studies suggest that NMES may induce effect on glucose metabolism similar to exercise training^36^. In the present study, we found that insulin sensitivity was improved after 10 sessions of NMES training compared to a sham-trained group of mice (Fig. 3). This metabolic effect was measured 48 h after NMES training suggesting that it was not an acute effect of NMES. The metabolic effect of electrical stimulation was suggested 30 years ago by Hoffman *et al*. and Roy *et al*., who both showed that NMES can stimulate signaling pathways involved in intracellular GLUT4 translocation in rats^27,29^. In addition, reduction of muscle atrophy has previously been related to improvement in glucose metabolism at the muscle level^37^. In fact, electrical muscle stimulation may locally prevent atrophy and improve glucose metabolism by increasing both p-AMPK and GLUT4 localization signaling pathways in critical illness myopathy^37^. In the present study, the NMES-induced increase in muscle mass may have led to an increase in glucose uptake at the muscle cells level, therefore improving insulin sensitivity^38^.

Other mechanisms of improvement in glucose uptake could be related to changes in blood flow or in sympathetic activity. Indeed, NMES may improve blood flow velocity at the site of stimulation^39^ and hence increase the rate of glucose extraction by muscle cells. Moreover, NMES has been reported to increase muscular sympathetic nerve activity^40^, that may, in turn, enhance whole body muscle glucose uptake^41^. These mechanisms could explain the results of the present study. However, the trend to a reduced blood pressure after training does not support these hypotheses, and, together with the relationship found between glucose uptake and change in muscle mass (Fig. 4), our results suggest that improvements in glucose uptake after NMES were primarily related to upregulation of muscle hypertrophy signaling pathways^24,25^. The lack of statistical significance is a limitation of the study, but this is likely a type B error since the p value would be 0.02 if sample size were doubled. The molecular mechanisms of action that allow local improvements to become systemic deserve to be further investigated. For example, it is now necessary to determine whether an increase in GLUT4 expression in skeletal muscle is responsible for the higher glucose uptake^42^ and, if so, whether it is related to the activation of the adenylate cyclase or to the IGF1/Akt signaling pathways^24^. Nevertheless, unilateral NMES is also known to improve electromyographic activity of the contralateral muscle in able-bodied individuals, suggesting that neural adaptations occur after NMES^32,43^. Hence, whether NMES also changes insulin secretion by acting on the central nervous system (such as the brain-endocrine pancreas axis)^44^ deserves to be further investigated.

Another important finding in the present study is that metabolic improvement induced by NMES might also transfer into cardiovascular changes. Associations between insulin resistance and hypertension have been described before^45^ and experimentally-induced decreases in insulin resistance have been associated with decreased blood pressure. For example, an antidiabetic agent enhancing insulin sensitivity can improve both glucose metabolism and blood pressure control in essential hypertensive patients with diabetes mellitus^46^. In addition, Improvements were significantly correlated to one another^46^. Although the long-term benefit of general exercise on both insulin sensitivity and blood pressure is well described^47^, the effect of short periods of training or local muscle strengthening on cardiometabolic outcomes is still unclear. In the present study, our results suggest that 2 weeks of NMES induce cardiovascular benefit via an improvement in insulin sensitivity. Enhanced endothelial nitric oxide synthesis^48^ or reduced renin-angiotensin-aldosterone system activity^49^ are potential mechanisms that could explain the cardiovascular improvement associated with enhanced insulin sensitivity. We previously reported that a short endurance training program improved both fasting glucose and systolic blood pressure in patients with COPD and that both improvements correlated with a reduction in pulse wave velocity, a surrogate marker of arterial stiffness^50^. Hence, our present results, yet preliminary, corroborate our findings in humans with chronic disease and suggest that even short periods of training can induce metabolic and possibly cardiovascular improvements. However, the effect of NMES on BP failed to be significantly different between groups. Repetitive anesthesia may have been a limiting factor to evaluate the hemodynamic effect of NMES. For example, 6 sessions of 30-min anesthesia with isoflurane can reduce heart rate and blood pressure in rodents^51^. Hence, repetitive anesthesia may have artificially reduced BP in both groups, therefore masking the actual effect of NMES on blood pressure. Additional studies are needed to confirm the effect of NMES on blood pressure in non-anesthetized animal models or in human.

Lastly, since long duration isoflurane exposure (~3 h) may also induce brain neurotoxicity and death in primates^52^, we expected potential death in our mouse population. However, only one mouse died during the training period. We cautiously reduced the impact of repetitive anesthesia by minimizing the total time of anesthesia for a given whole session of stimulation (25 min total anesthesia time for 20 min of NMES until the 5^th^ session, Fig. 1). However, the duration of both sleep latency and wake up increased after the 5^th^ session. This finding suggests that the mice have well tolerated 2.5 weeks of repetitive anesthesia but might have suffered hemodynamic repercussions (such as reduced BP), potentially limiting the application of NMES during a longer period (Figs 1 and 5). Although a 4-week period of NMES training with anesthesia seems to be feasible in mice^30^, there is very little information on the tolerance to anesthesia and potential death of animals in previous studies and further investigation is needed to determine if longer periods of training are well tolerated by mice.

Our results suggest that a model of chronic NMES training of only 2.5 weeks (12 sessions of 20 min) is sufficient to induce muscle adaptations in mice that transferred into improvements in insulin sensitivity. This metabolic effect of NMES might also transfer to cardiovascular benefit and could be of great utility for patients with chronic cardiometabolic disease unable to perform sufficient exercise training. However, our study revealed that repetitive anesthesia may have an impact on hemodynamics by reducing blood pressure over time, potentially dampening the actual effect of NMES on blood pressure. Nevertheless, this mouse model of chronic NMES training can be used to unravel the molecular mechanisms of NMES of local muscle strengthening/stimulation as an alternative to endurance training.

## Methods

### Animals

Twenty-four 16-week old male C57BL6 mice were used. Mice were randomly 1:1 assigned to NMES or sham training group. One mouse died in the NMES group. Therefore, NMES (n = 11) and sham (n = 12) were used for the analyses. Animals were housed in cage of 6, 3 per group, and were accustomed to living together two weeks prior to training. Alimentation and environment (space, light and noise) was strictly similar between cages. Tolerance to repetitive anesthesia and training sessions were carefully monitored. Mice had blood pressure measurements taken during the week before the beginning of training, and then before and after training. In addition, after training, mice underwent a metabolic test (intraperitoneal insulin tolerance test) and were sacrificed for muscle tissue collection and weighing.

The study was conducted in accordance with the European Convention for the Protection of Vertebrate Animals used for Experimental and Other Scientific Purposes (Council of Europe, European Treaties ETS 123, Strasbourg, 18 March 1986), and to the Guide for Care and Use of Laboratory Animals (NIH Publication No. 85–23, revised 1996). The protocol was approved by our local ethic comity (Grenoble Alpes University, 62-UTAH-HP2-MD-02, February 2016).

### Experimental set-up

**Figure 7:**
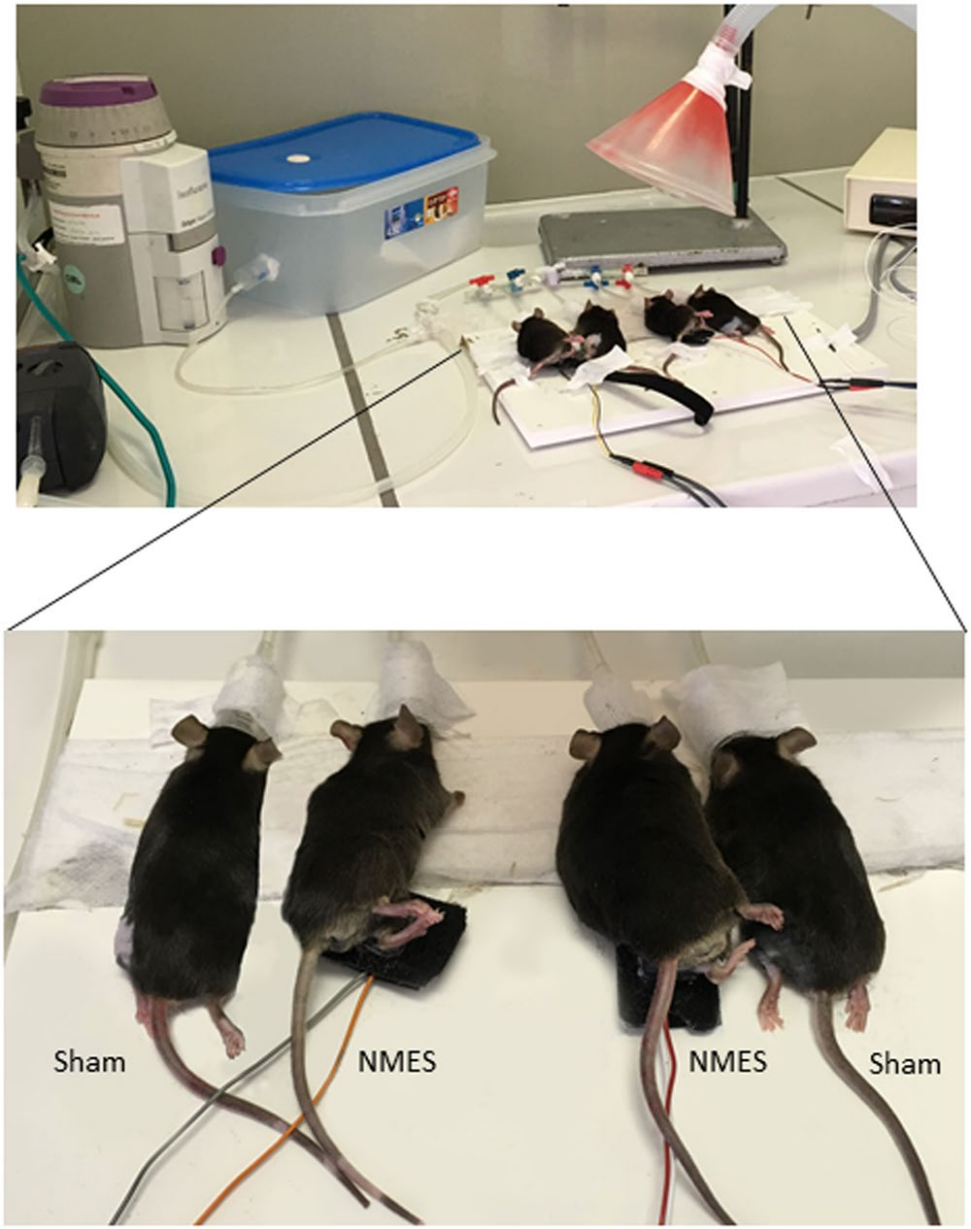
Study design. Mice were initially anesthetized through an induction chamber with isoflurane at 4% (blue box) before being continuously anesthetized during the NMES/sham training sessions with concentration of isoflurane regulated per awakening state of the mouse and respiratory movements (see Fig. 1 for isoflurane concentration).

The experimental set-up is presented on Fig. 7. One limb was designed to be stimulated/sham stimulated while the other was not. Since animal were lying in lateral recumbency, NMES/sham training sessions were performed on 4 mice simultaneously (2 NMES and 2 sham). Mice underwent 12 sessions × 20 minutes of NMES/sham training (5 times a week for 2.5 weeks) under anesthesia. A heating pad was used with a temperature probe (THEMTAB_V2 and THERM250_V2, Tem Sega, Pesac, France) to maintain normal core body temperature.

#### Anesthesia

Animals were anesthetized immediately before performing the NMES sessions using Isoflurane inhalation. Total time of anesthesia per day of stimulation was ~25 min (5 min for induction +20 min for training). An induction chamber with isoflurane at 4% was initially used. Then, isoflurane concentration was regulated according to sleeping/awakening state of the mouse and respiratory movements. The lowest percentage was used to minimize the risk of potential death^52,53^. Animals were alternatively breathing through one of the four isoflurane openings to ensure equal level of isoflurane between animals of the same group across the training period. Levels of isoflurane needed for the anesthesia during the training period are shown on Fig. 1. Air was mixed with oxygen at a constant flow of 1 L/min. A toe pinch was performed to ensure full anesthesia before the beginning of the NMES session.

#### Neuromuscular electrical stimulation

The NMES set-up was based on the previous works of Ambrosio *et al*.^30^. We used a clinical NMES device that was adapted for mouse by reducing the size of electrical cables and surface electrodes. Electrodes were built based on the model of Ambrosio *et al*.^30^. Briefly, the size of the NMES device wires was reduced via second copper wires connected to the former using a 2-way female wire connector, then allowing 2 wires winding pins spaced about 3.5 mm apart to be formed. Skin over the stimulated/sham-stimulated muscle was depilated before the training session and one another time during the training period (at the 8/9^th^ session) to ensure good electrical conduction of the current. Conducting gel was used between electrodes and skin. Electrodes were placed over the animal’s peroneal nerve, anterior to the fibular head. NMES consisted on electrically-induced ankle dorsiflexion using a symmetrical, biphasic, square-pulsed current (CEFAR Rehab4-Pro; CefarCompex) with a frequency of 50 Hz, a pulse duration of 150 μs and a duty cycle (on/off time) of 8/12 sec. A 1-second ramp up and down was used during the stimulation phase. These parameters were chosen because they matched previous clinical studies of our group demonstrating changes in muscle mass and insulin sensitivity after training^23,24^. The position of the electrodes and the effectiveness of NMES was checked visually (dorsiflexion and toes extension, see Fig. 7).

### Measurements

#### Trophic effect of NMES (Muscle tissue collection)

Tissue was collected 48 h after the last exercise session. Mice were euthanized by cervical dislocation. Lower limb muscles of both limbs including agonist muscles (TA, EDL), antagonist muscle (Gastroc.) and distal muscle (Quad.) were excised and weighed. The total muscle mass (TA + EDL + Gastroc + Quad) was compared between groups (NMES/sham) and conditions (stimulated/non -stimulated limb).

#### Metabolic effect of NMES (Intraperitoneal insulin tolerance test, ITT)

We assessed insulin sensitivity using the ITT as a marker of glucose uptake. We chose this test for its sensitivity to change in glucose uptake via the insulin pathway since we previously reported changes in muscle mass after NMES training via the insulin signaling pathway in vastus lateralis of patients with COPD^24^. Eight mice per group were used to perform this test. Tests were performed without anesthesia and 48 h after the end of the training period to avoid any potential impact of anesthesia on metabolic outcomes^54,55^. Mice were fasted for 5 hours then weighed before blood was collected from the tail tip for baseline glucose determination (t = 0) using the OneTouch® Ultra® glucometer. Insulin (0.5 IU/kg body weight, Novo Nordisk A/S) was injected intraperitoneally, followed by further blood glucose measurements at 15, 30, 45 and 60 minutes after the injection. Over the 60-minute period, the glucose nadir (the lowest blood glucose level) was calculated, and the glucose least square means was calculated for both groups.

#### Cardiovascular effect of NMES (non-invasive measurement of arterial pressure)

Blood pressure was measured using the mouse CODA tail-cuff system (Kent Scientific Corp, Torrington, CT). This validated technique consists in a cuff placed on the mouse’s tail to occlude blood flow and allow blood pressure measurements via a noninvasive blood pressure sensor placed distal to the occlusion cuff^56^ (Fig. 5a). After one week of acclimation (5 × 30 min/day), study measurements were performed one day before NMES training. Then, mice were kept acclimated to BP measurement once a week during the training period and were re-evaluated one day after the training period. For each mouse, multiple measurements of blood pressure were performed and averaged.

### Statistical analysis

Normality was assessed using Shapiro-Wilk tests. Between-group comparisons for average weight and isoflurane were performed using t-test or Mann Whitney Rank test (for non-parametric variables). Changes in body weight over time (before, after training) and between groups were assessed using a two-way analysis of variance (ANOVA). Differences in total muscle mass after training was assessed through a two-way analysis of variance (ANOVA) using group (NMES vs. sham) as Factor 1 and condition (stimulated vs. non-stimulated limb) as Factor 2. Differences in the stimulated limb to non-stimulated limb muscle mass ratio was assessed through a two-way analysis of variance (ANOVA) using group (NMES vs. sham) as Factor 1 and muscle type (TA, EDL, Gastroc, Quad) as Factor 2. A repeated measure analysis of variance was performed to compare i/the level of isoflurane across the training session or ii/the blood glucose concentration during the ITT, over time (factor 1), between groups (NMES vs. sham, factor 2). Lastly, a two-way repeated measure analysis of variance was performed to compare blood pressure (systolic, diastolic and mean BP) over time (before vs. after training, factor 1) between groups (NMES vs. sham, factor 2). Correlation coefficients were obtained with Pearson or Spearman (for non-parametric variables) coefficient correlations. Statistical significance was considered as p ≤ 0.05. Data are expressed as mean ± SE.

## Data Availability

The data are available per request to Isabelle Vivodtzev (ivivodtzev@partners.org).
